# IL-17A promotes *Helicobacter pylori*-induced gastric carcinogenesis via interactions with IL-17RC

**DOI:** 10.1007/s10120-022-01342-5

**Published:** 2022-09-20

**Authors:** Jee Hyun Kang, Suyoung Park, Jinhyung Rho, Eun-Ju Hong, Young-Eun Cho, Young-Suk Won, Hyo-Jung Kwon

**Affiliations:** 1 Department of Veterinary Pathology, College of Veterinary Medicine, Chungnam National University, 99 Daehak-ro, Yuseong-gu, Daejeon, 34134 Korea; 2 Department of Food and Nutrition, Andong National University, Andong, Korea; 3 Laboratory Animal Resource Center, Korea Research Institute of Bioscience and Biotechnology, Chungbuk, Korea

**Keywords:** Gastric cancer, *Helicobacter pylori*, IL-17A, IL-17RC, NF-κB, NOX1

## Abstract

**Background:**

Gastric cancer (GC) is a common malignancy worldwide, with a major attribution to *Helicobacter pylori*. Interleukin (IL)-17A has been reported to be up-regulated in serum and tumor of GC patients, but the precise mechanisms underlying its involvement in gastric tumorigenesis are yet to be established. Here, we investigated the roles of IL-17A in the pathogenesis of *H. pylori*-induced GC.

**Methods:**

GC was induced in IL-17A knockout (KO) and wild-type (WT) mice via *N*-methyl-*N*-nitrosourea (MNU) treatment and *H. pylori* infection. At 50 weeks after treatment, gastric tissues were examined by histopathology, immunohistochemistry, and immunoblot analyses. In vitro experiments on the human GC cell lines were additionally performed to elucidate the underlying mechanisms.

**Results:**

Deletion of IL-17A suppressed MNU and *H. pylori*-induced gastric tumor development accompanied by a decrease in gastric epithelial cell growth, oxidative stress, and expression of gastric epithelial stem cells markers. In AGS cells, recombinant human IL-17A (rhIL-17A) inhibited apoptosis and G1/S phase transition arrest while promoting reactive oxygen species production, sphere formation ability of cancer stem cells (CSC), and expression of stemness-related genes. In addition, rhIL-17A induced expression of IL-17RC, leading to NF-κB activation and increased NADPH oxidase 1 (NOX1) levels. Inhibition of NOX1 with GKT136901 attenuated rhIL-17A-mediated elevation of GC cell growth, ROS generation, and CSC stemness. Clinically, IL-17RC expressions were significantly upregulated in human GC compared with normal gastric tissues.

**Conclusion:**

Our results suggest that IL-17A promotes gastric carcinogenesis, in part, by regulating IL-17RC/NF-κB/NOX1 pathway, supporting its potential as a target in human GC therapy.

**Supplementary Information:**

The online version contains supplementary material available at 10.1007/s10120-022-01342-5.

## Background

Gastric cancer (GC) is one of the most prevalent malignant cancer types worldwide and particularly widespread in countries of Eastern Asia [1, 2]. While the etiology of GC is thought to be multifactorial, *Helicobacter pylori* infection has been identified as a major risk factor. *H. pylori* infection induces chronic gastric inflammation, which can progress to chronic atrophic gastritis, intestinal metaplasia and dysplasia, and ultimately, GC [3, 4]. *H. pylori* interactions with the gastric mucosa trigger several key events linked to pathogenesis, specifically, (1) an inflammatory response with the release of various cytokines and reactive oxygen species (ROS), (2) glandular atrophy following long-term infection and interaction with host responses, and (3) cellular proliferative changes, such as dysplasia and metaplasia [5]. Colonization by *H. pylori* leads to the recruitment of immune cells, including neutrophils, M1 and M2 macrophages, and T and B lymphocytes, to the stomach. Immune cells secrete cytokines, which directly or indirectly damage surface epithelial cells, leading to loss of microvilli, irregularity of the luminal border and vacuolation. Oxidative stress is another hallmark of *H. pylori* infection and increased levels of ROS are typically observed in gastric epithelial cells [6]. Excessive production of ROS is believed to be a major cause of altered epithelial cell proliferation and DNA damage in infected gastric mucosa, in turn, promoting genomic instability and tumorigenesis [7].

Interleukin (IL)-17 is a relatively novel family of critical inflammatory cytokines consisting of six family members designated IL-17A-F [8]. IL-17A is a pro-inflammatory cytokine secreted by CD4^+^ T helper 17 cells (Th17), along with other immune cells such as CD8^+^ T cells, natural killer cells, and γ-δ T cells [9]. IL-17 plays key role in several inflammatory diseases and is proposed to be involved in inflammation-related tumor formation [10–12]. Upregulation of IL-17A positively correlated with the severity of gastritis has been reported in biopsies of *H. pylori*-infected individuals relative to uninfected controls [13]. Genetic polymorphisms of IL-17A are associated with susceptibility to GC, especially in cases of *H. pylori* infection [14]. Moreover, expression of IL-17A is significantly increased in tumors of GC patients relative to adjacent nontumor gastric mucosa [15, 16]. Increased expression of IL-17A has additionally been reported in sera of GC patients compared to healthy controls [17–19]. However, studies to date on the role of IL-17A in GC development have generated inconsistent findings. For instance, IL-17A has been shown to promote tumor growth by stimulating angiogenesis and invasive capacity of tumor cells along with inhibiting apoptosis [20–22]. In contrast, infiltration of intratumoral IL-17A- producing cells is correlated with antitumor immune contexture and improved response to adjuvant chemotherapy in GC [15]. In the present study, we have addressed the role of IL-17A in *H. pylori*-associated gastric carcinogenesis and the precise mechanisms underlying its contribution to tumor development using an *H. pylori*-induced mouse gastric tumor model and human GC cell lines.

## Materials and methods

### Induction of gastric tumors in mice

Induction of GC in mice was performed as described previously [23]. Five-week-old male IL-17A KO and wild-type (WT) mice with a C57BL/6 background were randomly divided into four groups (Supplementary Fig. 1). Animals in groups 1 and 2 were administered *N*-methyl-*N*-nitrosourea (MNU; 150 ppm) every other week for 10 weeks. One week after completion of MNU treatment, mice were inoculated with *H. pylori* three times every other day. Groups 3 and 4 were fed a standard pellet chow diet. Mice were sacrificed 38 weeks after infection, making a total of 50 weeks of treatment (Supplementary Fig. 1).

### Statistical analysis

All data are expressed as means ± standard error of the mean. Statistical analyses were performed using GraphPad Prism software (GraphPad, CA, USA). Correlations between IL-17RC expression and clinicopathological features were analyzed using the Chi-square test. The incidence of gastric tumors and gastric histologic scores were evaluated with Fisher’s exact or Mann–Whitney *U* test, respectively. Other data were compared using unpaired two-tailed Student’s t-test and ANOVA. *P* values < 0.05 were considered statistically significant.

## Results

### IL-17A depletion suppresses development of H. pylori-induced GC

**Fig. 1:**
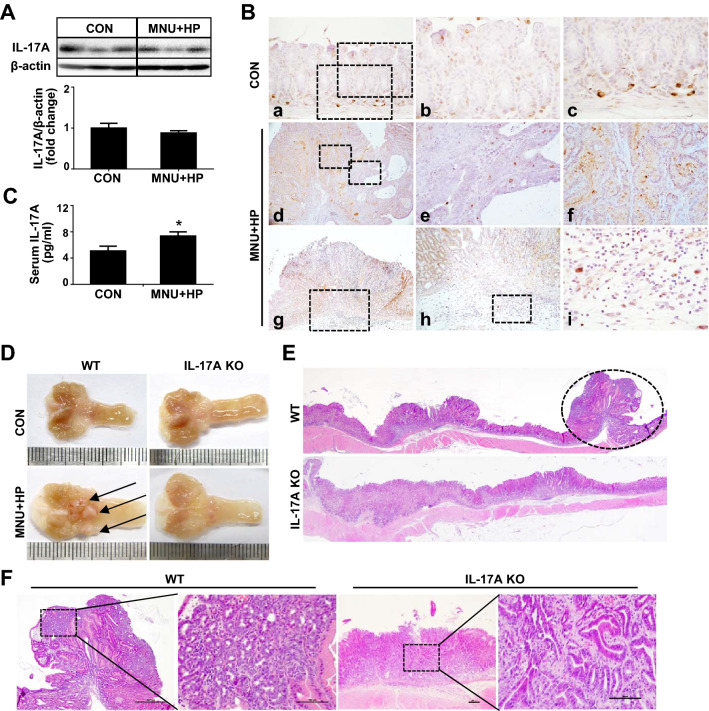
IL-17A deficiency suppresses *H. pylori*-induced gastric cancer. **A** Western blot analysis of IL-17A in gastric tissues of control and MNU and *H. pylori* treated WT mice. **B** Immunoreactivity of IL-17A in stomach tissues of control and MNU and *H. pylori*-treated WT mice. The low magnification (X200) of gastric tissues of control mice (**a**). The high magnification (X400) of (**a**) shows surface mucous (**b**) and gastric glands (**c**). The low magnification (X40) of gastric cancer of MNU and *H. pylori*-treated WT mice (**d**). The high magnification (X400) of (**d**) shows tumor (**e**) and adjacent non-tumor (**f**) lesions. No IL-17A positivity was observed in tumor cells, but strong immunoreactivity was found in proliferating and metastatic gastric cells. The low magnification (X40) of the stomach of MNU and *H. pylori*-treated WT mice (**g**). The high magnification of (**g**) shows submucosa (X100, **h**) and infiltration of inflammatory cells (X400, **i**). Note the strong IL-17A immunoreactivity in the cytoplasm of inflammatory cells. **C** Serum concentrations of IL-17A in control and MNU and *H. pylori-*treated WT mice. **D** Macroscopic images of control and MNU/*H. pylori-*treated stomach tissues of WT and IL-17A KO mice. Growth of polypoid to sessile masses pinkish in color (arrows) in stomachs of MNU and *H. pylori-*treated WT mice. **E** Hematoxylin and eosin **H** and **E** staining of MNU and *H. pylori-*treated WT and IL-17A KO stomach tissues. Neoplastic nodules (circles) showing a polypoid appearance in the antrum (original magnification =  ×10). **F** Magnification of neoplastic nodules. Boxed regions of left panels (original magnification = ×40) are shown at a higher magnification (original magnification = ×200) in the right panels. Nodules in WT mouse stomach consist of pleomorphic tumor cells showing a sessile growth pattern, with invasion to submucosa. Nodules in IL-17A KO mouse stomach show glandular and tubular growth patterns composed of well-differentiated tumor cells. Data are expressed as mean ± SEM. *P < 0.05 versus control

Previous studies have shown higher levels of IL-17A in serum and cancer tissues of patients with GC compared with healthy controls [15–19]. Moreover, elevated IL-17A expression in gastric mucosa of mice infected with *H. pylori* has been reported [24]*.* We initially evaluated IL-17A expression in gastric tumors of WT mice infected with *H. pylori*. As shown in Fig. 1A, no significant differences in stomach IL-17A levels were observed between control and MNU and *H. pylori*-treated mice. Immunohistochemical analysis revealed IL-17A expression in surface mucous epithelium and gastric glands of WT mouse stomach. After MNU and *H. pylori* treatment, immunoreactivity for IL-17A was detected in the cytoplasm of inflammatory cells as well as surface mucous epitheliums and gastric glands, but not tumor cells (Fig. 1B). Co-immunofluorescence staining showed IL-17A-positive cells were mainly expressed in CD4^+^ and CD8^+^ cells (Supplementary Fig. 2A) and MNU and *H. pylori*-treated mice showed a higher number of CD4^+^ and CD8^+^ cells than control mice (Supplementary Fig. 2B). In addition, serum IL-17A was significantly elevated in MNU and *H. pylori*- treated mice compared with control (Fig. 1C), suggesting an association of serum IL-17A with *H. pylori*-induced tumor development in our model.

To further explore the role of IL-17A in *H. pylori*-induced gastric carcinogenesis, IL-17A KO mice and WT littermates were treated with MNU and *H. pylori* and their stomachs examined at the end of week 50. Tumors developed mostly in the pyloric mucosa adjacent to the fundic region in both WT and IL-17A KO mice (Fig. 1D, E). Macroscopically, most tumors exhibited a polypoid growth pattern similar to that of type I stomach cancers in humans and some were sessile (Fig. 1D). Histopathologically, the overall incidence of stomach tumors was lower in IL-17A KO than WT mice (Supplementary Table 2). Although no significant differences in gastric adenoma incidence were evident, the frequency of gastric adenocarcinoma was markedly decreased in IL-17A KO mice compared with WT mice (Supplementary Table 2). Tumor multiplicity was also lower in IL-17A KO than in WT mice (Supplementary Table 2). Neoplastic nodules from WT mice were composed of pleomorphic neoplastic cells exhibiting moderate to poor differentiation that formed irregular tubules or solid nests (Fig. 1F). In contrast, neoplastic nodules from IL-17A KO mice comprised small acini and tubules composed of well-differentiated neoplastic cells (Fig. 1F). Our results indicate that lack of IL-17A suppresses the development of *H. pylori*-induced GC, supporting its role as an important tumor-promoting factor in this model.

In addition to tumor classification, preneoplastic lesions were investigated. Varying degrees of preneoplastic lesions, such as inflammation, foveolar hyperplasia, intestinal metaplasia, and dysplasia, were observed in mice exposed to MNU and *H. pylori* (Supplementary Fig. 3). All lesions were significantly milder in IL-17A KO mice relative to their WT counterparts (Supplementary Fig. 3). In addition, IL-17A KO mice exhibited a lower number of Ly6G-positive cells than WT mice (Supplementary Fig. 4), suggesting that IL-17A depletion suppresses the progression of multistep carcinogenesis of GC.

### IL-17A regulates proliferation and apoptosis of gastric epithelial cells

**Fig. 2:**
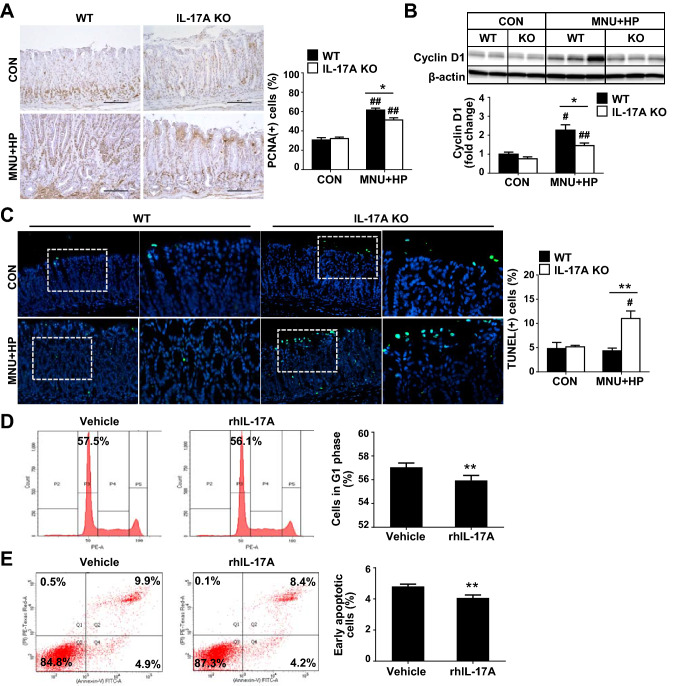
IL-17A regulates growth of gastric epithelial cells. **A** Immunohistochemical analysis of proliferating cell nuclear antigen (PCNA) of gastric tissues of WT and IL-17A KO mice (original magnification = ×200). Ten random fields of each sample were selected and positive cell in each image were counted. **B** Immunoblot analysis of cyclin D1 in WT and IL-17A KO mice. Expression of cyclin D1 is normalized to that of β-actin. **C** Representative photomicrographs of TUNEL staining in gastric tissues of WT and IL-17A KO mice. Boxed regions of left panels (original magnification = ×200) are shown at higher magnification (original magnification = ×400) in the right panels. Ten fields were randomly selected for the quantification of positive cells per slide. **D** Cell cycle analysis. AGS cells were treated with rhIL-17A (50 ng/ml) for 12 h and changes in the percentage of G1 phase of cells were measured via propidium iodide (PI) staining using flow cytometry. **E** Analysis of cell apoptosis. The degree of apoptosis was assessed via flow cytometry using Annexin V and PI staining. Data are presented as mean ± SEM. *P < 0.05, **P < 0.01 versus WT or vehicle; ^#^*P* < 0.05, ^##^*P* < 0.01 versus same genotype control

The integrity of the normal gastric mucosa is maintained by controlling the balance of cell numbers via proliferation and apoptosis. Excessive cell proliferation greater than the level of cell death results in the development of malignant neoplasms [25]. Accordingly, we analyzed the proliferation and apoptosis of gastric epithelial cells in non-cancerous stomach samples. As shown in Fig. 2A, MNU and *H. pylori*-treated IL-17A KO mice displayed a lower number of PCNA-positive cells than WT mice. Consistent with these results, expression of cyclin D1 was decreased in IL-17A KO relative to WT mice (Fig. 2B). Conversely, the number of TUNEL-positive cells was significantly higher in the surface epithelium of gastric mucosa of IL-17A KO mice exposed to MNU and *H. pylori* (Fig. 2C). To further investigate the potential role of IL-17A in proliferation and apoptosis, AGS cells were treated with rhIL-17A. Flow cytometry analysis exhibited a significantly lower percentage of cells at the G1 phase in rhIL‑17A-treated AGS compared to vehicle-treated cells (Fig. 2D), suggesting that IL-17A promotes the transition of the cell cycle from the G1 to S phase. In addition, the percentage of early apoptotic cells was significantly decreased in rhIL‑17A-treated AGS compared with vehicle-treated cells (Fig. 2E). These results indicate that IL-17A promotes proliferation while inhibiting apoptosis of GC cells.

### IL-17A promotes H. pylori-induced ROS production

**Fig. 3:**
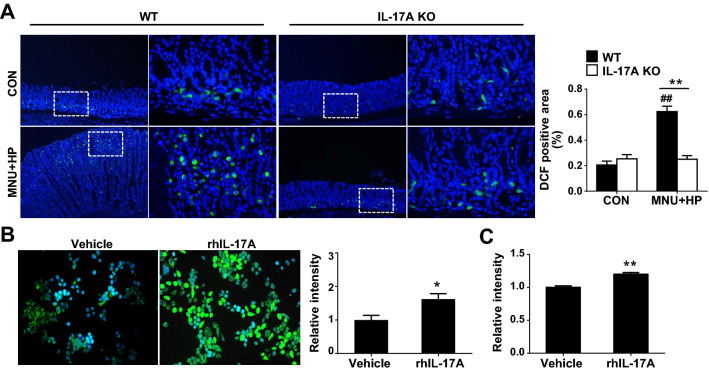
IL-17A increases *H. pylori*-induced oxidative stress. **A** Representative images of DCF (green) and 4′,6-diamidino-2-phenylindole (DAPI; blue) staining in gastric tissues of WT and IL-17A KO mice (original magnification = ×100 (left) and ×400 (right)). Five fields were randomly selected for each section and the positive area in each image was measured using ImageJ software. **B** ROS levels in AGS cells. AGS cells were treated with rhIL-17A (50 ng/ml) or vehicle for 12 h and stained with DCFH. **C** ROS production in AGS cells. AGS cells were treated with rhIL-17 (50 ng/ml) and DCF fluorescence was detected using a spectrophotometer. Data are presented as mean ± SEM. *P < 0.05, **P < 0.01 versus WT or vehicle; ^#^*P* < 0.05, ^##^*P* < 0.01 versus same genotype control

*H. pylori*-induced oxidative stress plays a critical role in the inflammation of the gastric mucosa and enhances the risk of GC development [26]. As shown in Fig. 3A, DCF-positive cells were rarely detected in the gastric gland base in both control WT and IL-17A KO mice. Treatment of MNU and *H. pylori*-induced an increase in the number of DCF-positive cells, but to a significantly lower extent in IL-17A KO than in WT mice (Fig. 3A). To further determine the direct effects of IL-17A on oxidative stress of GC, intracellular ROS levels were analyzed in AGS cells treated with DCFH fluorescent dye using fluorescence microscopy and spectrofluorometry. DCF fluorescence in AGS cells exposed to rhIL-17A was higher relative to that in vehicle-treated cells (Fig. 3B). Consistently, spectrofluorometry analysis showed significantly higher production of ROS in AGS cells exposed to rhIL-17A than vehicle-treated cells (Fig. 3C), indicating that IL-17A increases oxidative stress in GC cells.

### IL-17A increases stemness of GC cells

**Fig. 4:**
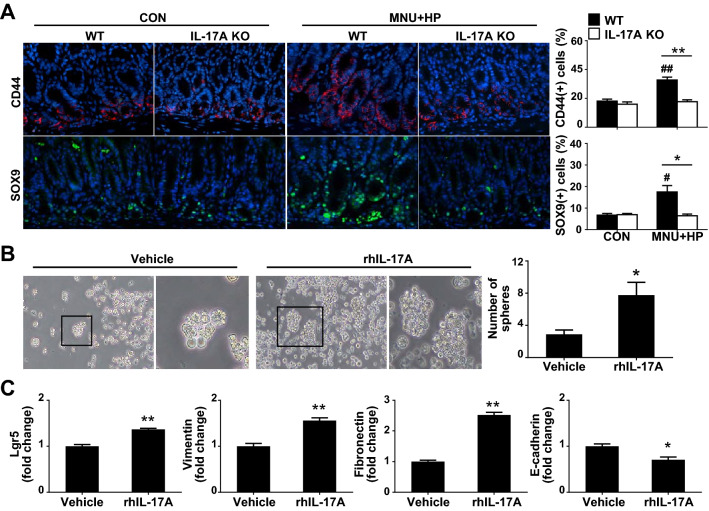
IL-17A promotes the stemness of gastric cancer cells. **A** Immunofluorescence imaging of CD44 (red) and SOX9 (green) in stomach tissue of WT and IL-17A KO mice. In the gastric epithelium, CD44- and SOX9-positive cells of a total of 100 cells were counted, respectively (original magnification = ×400). **B** Sphere forming assay. AGS cells were cultured in conditioned stem cell medium for 4 weeks and stimulated with rhIL-17A (50 ng/ml) or vehicle. Spheres containing ≥ 10 cells were counted per well of a 24-well plate. Boxed regions of left panels (original magnification = ×40) are shown at higher magnification (original magnification = X100) in the right panels. **C** Real-time PCR analysis of Lgr5, vimentin, fibronectin, and E-cadherin in AGS cell-derived spheres treated with rhIL-17A or vehicle. Data are expressed as the mean ± SEM. *P < 0.05, **P < 0.01 versus WT or vehicle; ^#^*P* < 0.05, ^##^*P* < 0.01 versus same genotype control

Cancer growth is driven by a rare subpopulation of CSCs with self-renewal ability that can induce tumor proliferation, formation of spheroids and tumorigenesis [27]. Epithelial-mesenchymal transition (EMT) in cancer cells facilitates the acquisition of mesenchymal traits and expression of stem cell markers [28]. To clarify whether decreased development of *H. pylori*-induced GC in IL-17A KO mice is associated with CSCs, we examined the expression levels of CD44 and SOX9, CSC stemness-related surface markers. As shown in Fig. 4A, CD44- and SOX9-positive cells were significantly increased in the base of gastric glands of MNU and *H. pylori*-treated WT mice compared with their control WT counterparts. Notably, MNU and *H. pylori*-exposed IL-17A KO mice contained a lower proportion of CD44- and SOX9-positive cells than treated WT mice (Fig. 4A). To further ascertain the effects of IL-17A on stemness of GC cells, AGS cells were treated with rhIL-17A in conditioned stem cell medium. Compared with vehicle-treated cell, rhIL-17A significantly promoted sphere formation ability (Fig. 4B). In addition, expression levels of Lgr5, vimentin, and fibronectin were markedly higher in rhIL-17A-treated AGS than vehicle-treated cells whereas the E-cadherin content was decreased (Fig. 4C). Our findings suggest that IL-17A promotes tumor progression through regulating stemness of GC cells.

### *IL-17A enhances H. pylori-induced gastric carcinogenesis *via* IL-17RC/NF-kB/NOX1 pathway*

**Fig. 5:**
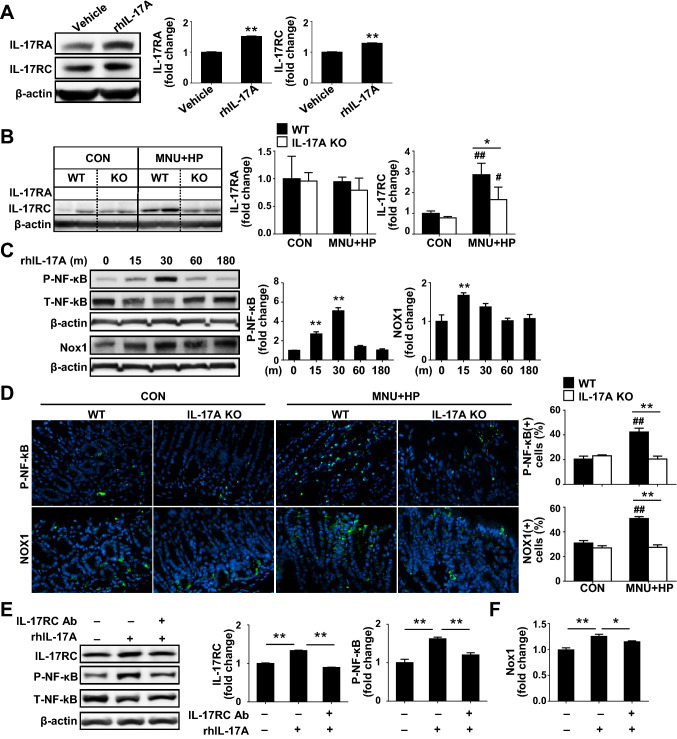
IL-17A regulates *H. pylori*-induced GC development via IL-17RC/NF-kB/NOX1 pathway. **A** Western blot analysis of IL-17RA and IL-17RC in rhIL-17A-treated AGS cells. **B** Expression of IL-17RA and IL-17RC in MNU and *H. pylori-*treated WT and IL-17A KO mice. **C** Western blot analysis of phospho-NF-κB and NOX1 in rhIL-17A-treated AGS cells. **D** Immunofluorescence images of phospho-NF-κB and NOX1 (green) in stomach tissue of WT and IL-17A KO mice. Positive cells of phospho-NF-κB and NOX1 were counted in ten randomly selected fields (original magnification = ×400). **E** Western blot analysis of the levels of the IL-17RC and phospho-NF-κB in rhIL-17A and/or anti-IL-17RC-treated AGS cells. **F** Real-time PCR analysis of NOX1 in AGS cells treated with rhIL-17A and/or anti-IL-17RC. Data are expressed as mean ± SEM. *P < 0.05, **P < 0.01 versus WT or vehicle; ^#^*P* < 0.05, ^##^*P* < 0.01 versus same genotype control

IL-17A exerts its effects through binding to receptors IL-17RA and IL-17RC, leading to the activation of intracellular signaling pathways [29]. To elucidate the mechanisms by which IL-17A promotes *H. pylori*-induced gastric carcinogenesis, we analyzed the expression patterns of IL-17RA and IL-17RC. *H. pylori* infection led to an increase in IL-17RA and IL-17RC levels in time- and dose-dependent manner in AGS cells (Supplementary Fig. 5A). Treatment with rhIL-17A also induced elevation of IL-17RA and RC levels in AGS and SNU 601 cells (Fig. 5A, Supplementary Fig. 5B). Interestingly, MNU and *H. pylori* treatment had no effect on the IL-17RA levels in both WT and IL-17A KO mouse groups (Fig. 5B). In contrast, IL-17RC expression was significantly increased following MNU and *H. pylori* treatment, but to significantly lower extent in IL-17A KO than WT mice (Fig. 5B). We further analyzed whether decreased CSC stemness in MNU and *H. pylori*-exposed IL-17A KO mice is related to IL-17RC via immunofluorescence staining of stomach samples. IL-17RC clearly co-localized with CD44 in gastric glands of MNU and *H. pylori*-treated mice, but to a lower extent in the IL-17A KO than the WT group (Supplementary Fig. 5C).

To further elucidate the mechanisms by which IL-17A/IL-17RC signaling promotes gastric carcinogenesis, we examined the NF-κB/NADPH oxidase (NOX) signal pathway. *H. pylori* infection triggers activation of NF-κB in the stomach, which induces expression of various target, such as NOX1 [30, 31]. The NOX family, a source of intracellular ROS, is classified into seven NOX homologs (NOX1-5, dual oxidase (DUOX) 1, and DUOX2) [32]. Treatment with rhIL-17A led to enhanced NF-κB activation in AGS and SNU601 cells (Fig. 5C, Supplementary Fig. 6A). Moreover, *NOX1, NOX2, NOX5,* and *DUOX2* mRNA expression in AGS cells was significantly increased in a time-dependent manner in the presence of rhIL-17A (Supplementary Fig, 6B). Western blot analysis further confirmed that rhIL-17A treatment induced an increase in NOX1 levels in both AGS and SNU601 cells (Fig. 5C, Supplementary Fig. 6A). Consistent with in vitro results, phospho-NF-κB and NOX1-positive cells were significantly increased in MNU and *H. pylori*-exposed WT relative to control WT mice. However, MNU and *H. pylori*-treated IL-17A KO mice showed a lower number of phospho-NF-κB and NOX1-positive cells than WT mice (Fig. 5D). In addition, the immunofluorescence staining showed that phospho-NF-κB co-localized with NOX1 in the stomach (Supplementary Fig. 7). To confirm the effects of IL-17A/IL-17RC on the NF-κB/NOX1 pathway, rhIL-17A-treated AGS cells were incubated with an IL-17RC neutralizing antibody. Blockage of IL-17RC suppressed IL-17A-induced NF-κB activation and NOX1 expression (Fig. 5E, F). Moreover, suppression of NF-κB with a specific inhibitor, IMD-0354, led to the downregulation of the IL-17A-induced increase in NOX1 expression (Supplementary Fig. 6C, D). These data clearly indicate that IL-17A/IL-17RC regulates GC development through modulation of the NF-κB/NOX1 signaling pathway.

### Inhibition of NOX1 suppresses IL-17A-induced GC cell growth, oxidative stress, and CSC stemness

**Fig. 6:**
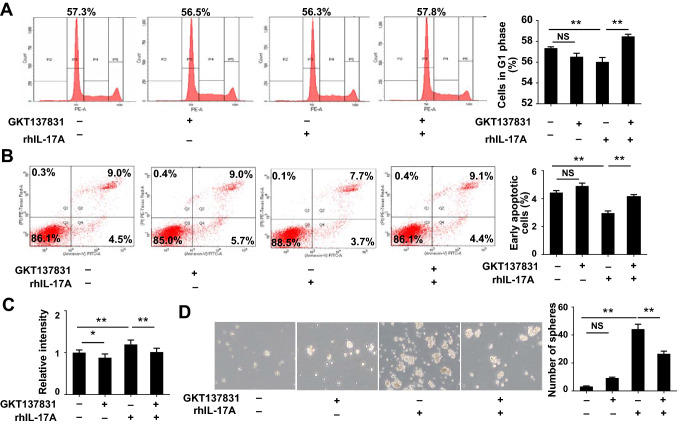
Inhibition of NOX1 attenuates IL-17A-induced epithelial cells growth, oxidative stress, and stemness of gastric cancer cells. **A** Cell cycle analysis of AGS cells treated with rhIL-17A and/or GKT137831. **B** Changes in percentages of apoptotic cells treated with rhIL-17A and/or GKT137831. **C** ROS levels in AGS cells treated with rhIL-17A and/or GKT137831. **D** Sphere formation analysis in AGS-derived spheres treated with rhIL-17A and/or GKT137831. Twelve fields were randomly selected for quantification of spheres per 3 wells of a 6-well plate (original magnification = ×40). Data are presented as mean ± SEM. *P < 0.05, **P < 0.01

Given the critical role of NF-κB/NOX1 in IL-17A/IL-17RC signaling, we examined the hypothesis that NOX1 inhibition could suppress the IL-17A-induced increase in gastric carcinogenesis. As shown previously by our group, rhIL-17A treatment of AGS cells resulted in a reduced percentage of PI‑stained cells in the G1 phase (Fig. 6A). Treatment with GKT136901, a NOX1 specific inhibitor, increased the percentage of cells in the G1 phase, indicating that NOX1 inhibition blocks transition of the cell cycle from the G1 to S phase induced by rhIL-17A. In addition, the rhIL-17A-induced decrease of apoptosis was reversed by GKT136901 in AGS cells (Fig. 6B), along with a reduction of ROS production and sphere formation ability (Fig. 6C, D). The treatment of GKT136901 alone down-regulated ROS production in AGS cells (Fig. 6C), but no significant difference was observed in cells in G1 phase, apoptotic cells, and a number of spheres (Fig. 6). These findings collectively support a pivotal role of the NF-κB/NOX1 pathway in IL-17A/IL-17RC-induced GC development.

### IL-17RC is upregulated in human GC tissues

**Fig. 7:**
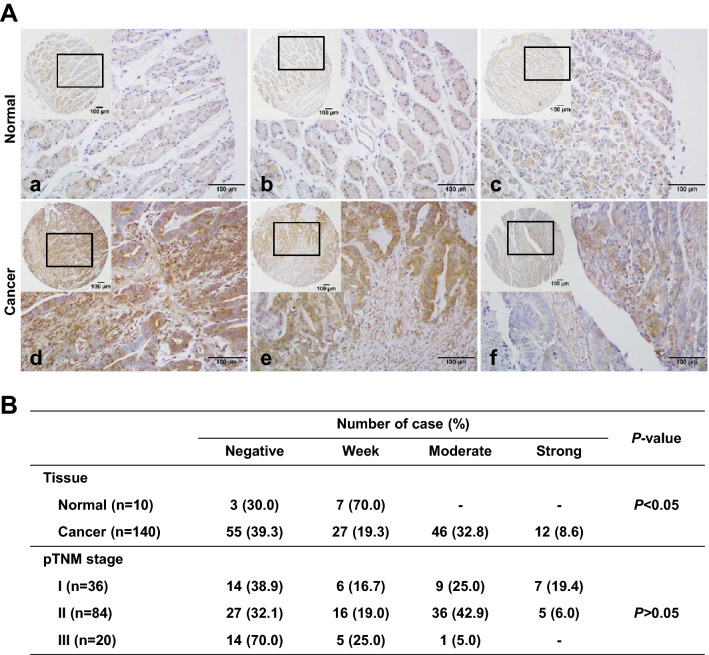
Immunohistochemical (IHC) staining for IL-17RC in human gastric cancer and normal tissue samples. **A** Representative photomicrographs of IHC for IL-17RC. Note a weak positive IL-17RC staining of normal gastric tissue. Gastric cancer tissues showed strong (d; TNM stage Ia), moderate (e; TNM stage II), and weak (f; TNM stage III) IL-17RC expressions. Original magnification = ×40 or ×400. **B** Correlation between IL-17RC expression and tissue types and pTNM stage

Using human microarray slides, we compared the degrees of IL-17RC expression in gastric cancer (tumor) and normal (non-tumor) tissues. IL-17RC expression was significantly higher in gastric cancer than in normal tissues (Fig. 7A). In cancer tissues, 41.4% of samples showed moderate to strong positive IL-17RC expression whereas 100.0% of normal tissues displayed negative to weak expression (Fig. 7A). However, the degree of IL-17RC expression in human gastric cancer tissues was not correlated with TNM stage (Fig. 7B).

## Discussion

Several human clinical studies have established a relationship between the expression of IL-17A and GC progression. Upregulation of IL-17A is observed in serum and tumor samples of GC patients compared to healthy controls [17–21]. In the present study, IL-17A protein was detectable in the surface mucous epithelium and gastric glands of a normal WT mouse stomach. After MNU and *H. pylori* treatment, immunoreactivity for IL-17A was present in inflammatory as well as surface mucous cells and gastric glands, but not tumor cells. This finding is consistent with a previous report that tumor cells themselves do not express IL-17A [16]. Wu et al. [16] showed higher expression of IL-17A protein in GC tissues than in adjacent nontumor gastric mucosa. However, immunoreactivity for IL-17A was observed mainly in cytoplasm of intratumoral inflammatory cells but not tumor cells. The group also reported no IL-17A mRNA expression in a variety of GC cell lines although IL-17A protein levels were increased in the serum of GC patients compared to healthy controls. Consistent with these findings, our data showed significantly higher serum IL-17A levels in MNU and *H. pylori*-treated than control mice, supporting the involvement of IL-17A in *H. pylori* infection and tumor development in our model. Furthermore, immunoblotting exhibited the expression of IL-17A in stomach was not different between control and MNU and *H. pylori*-treated mice, but, the inflammatory cells, mainly CD4^+^ and CD8^+^ cells expressed IL-17A and these expression levels was more prominent in MNU and *H. pylori*-treated mice compared with control. IL‑17A production has been found in CD4^+^ T and CD8^+^ T (Tc17) cells [9]. Although these results could not explain directly the role of IL-17A^+^ inflammatory cells in tumor development, increased GC development in MNU and *H. pylori*-treated mice was associated with an elevated number of CD4^+^ and CD8^+^ cells.

While IL-17A is clearly increased in human gastric tumors, its specific contribution to tumor pathogenesis remains elusive. IL-17A is reported to be involved in tumor cell migration and invasion, chemotherapy resistance, and immunosuppression, promoting cancer progression and metastasis [33–35]. Wang et al*.* [22] showed that IL-17A enhances migration and invasiveness of GC cells via activation of NF-kB pathway and subsequent upregulation of MMP-2 and MMP-9, consistent with earlier reports that GC cells or tumor-associated neutrophils produce IL-17A, which is associated with EMT [20, 21]. In contrast, several studies have described opposite results, i.e., antitumor and anti-*H. pylori* effects of IL-17A. For instance, higher IL-17A^+^ cells infiltration is reported to be correlated with improved survival and enriched intratumoral IL-17A^+^ cells, indicative of an antitumor immune contexture in GC [15]. Additionally, an anti-inflammatory effect of IL-17A on *H. pylori*-induced gastritis through suppression of Th1 differentiation and bacterial colonization has been observed [24, 36]. In the present study, GC development induced by MNU and *H. pylori* infection was significantly suppressed under conditions of IL-17A deficiency. Additionally, IL-17A depletion attenuated the degrees of chronic gastritis and preneoplastic lesions, clearly indicating an oncogenic role in GC development.

Reduced incidence of GC in IL-17A KO mice in our experiments was correlated with decreased inflammation, GC cell growth, ROS production, and GC cell stemness. Several studies show that IL-17A promotes the activation and accumulation of inflammatory cells, such as neutrophils, which can promote the initial stages of tumorigenesis [37, 38]. IL-17A is also involved in the growth of multiple tumor types and oxidative stress. For instance, IL-17A can directly induce tumor cell proliferation and suppress apoptosis of GC cells and diffuse large B cell lymphoma cells [16, 39]. Moreover, IL-17A is reported to cause an increase in intracellular ROS in esophagus adenocarcinoma and vascular smooth muscle cells [40, 41]. In addition, IL-17 family members possess potentially crucial biological activities that mediate CSC progression. For instance, IL-17 promotes the self-renewal of CD133^+^ cancer stem-like cells in ovarian cancer [42] and facilitates the transformation of quiescent to invasive gastric CSCs [33]. IL-17B can enhance the stemness of GC cells and contribute to self-renewal and tumorigenesis of CSCs [43]. In our experiments, IL-17A KO mice exhibited lower expression of Ly6G-positive cells, gastric epithelial cell growth, oxidative stress, and expression of gastric epithelial stem cells markers. In addition, exogenous rhIL-17A directly promoted the growth of GC cells, ROS production, sphere formation ability of CSCs, and expression of stemness-related genes. While current knowledge of the biological functions of IL-17A in GC remains limited, our results support its potential utility as a novel target for GC.

IL-17A signaling is mediated by a heterodimeric receptor complex consisting of the IL-17RA and IL-17RC. Several clinical studies have demonstrated an essential role of both IL-17RA and IL-17RC in cancer progression, including prostatic and lung cancer and GC [44–46]. *Jiang *et al*.* [46] reported overexpression of IL-17A in GC tissues compared with adjacent normal tissues, which was correlated significantly with tumor progression, lymphatic invasion, and metastasis. In addition, downregulation of either IL-17RA or IL-17RC in GC cells led to complete abrogation of the intracellular signal transduction pathway activated by IL-17A [47]. In our study, only IL-17RC expression was suppressed in MNU and *H. pylori* treated IL-17A KO compared with WT mice whereas no difference in IL-17RA levels were observed. These results are consistent with those of a previous study showing significantly elevated IL-17RC but not IL-17RA expression in prostatic tissue from benign prostatic hyperplasia and prostate cancer patients compared with controls [48]. While IL-17RA and IL-17RC subunits operate in concert to mediate IL-17A signaling, IL-17RC possesses unique intracellular domains involved in the modulation of IL-17A-induced signaling [49]. Given that IL-17RA and IL-17RC are differentially expressed by hematopoietic and non-hematopoietic cells [50], the IL-17RA/IL-17RC ratio is postulated to regulate IL-17A-induced cell response in a cell type-dependent manner. Interestingly, tissue microarrays of human stomach cancer samples clearly revealed increased expression of IL-17RC in GC compared with the normal stomach. The present study not only confirmed the upregulation of IL-17RC in human GC but also provided insights into potential candidate mechanisms underlying the regulatory effects of IL-17A/IL-17RC in gastric carcinogenesis. However, we have not investigated how *H. pylori* and IL-17A induce increased expression of IL-17RC in GC cells. Additional research is needed to evaluate the signaling pathway downstream to IL-17RC induced by *H. pylori* and IL-17A in GC.

We further established the NF-κB/NOX1 signaling axis as a specific pathway implicated in IL-17A/IL-17RC-mediated regulation of GC development. In human GC, constant activation of NF-κB is one of the key early events in neoplastic progression [51]. NF-κB is capable of inducing NOX1 expression and the generation of NOX-derived ROS is dependent on NF-kB activity in colon cancer cells [52]. In addition, NOX1 expression is elevated in human *H. pylori*-associated gastritis and GC [53] and *H. pylori* induces NOX1-derived ROS in guinea pig gastric mucosal cells [54]. In the present study, NF-κB activation and NOX1 expression were suppressed in the stomach of MNU and *H. pylori*-treated IL-17A KO mice. In addition, rhIL-17A treatment enhanced NF-κB phosphorylation and NOX1 expression in GC cells and these effects were significantly abrogated upon disruption of IL-17RC. rhIL-17A-induced NOX1 upregulation was suppressed by an NF-κB inhibitor, indicating that IL-17A promotes gastric carcinogenesis through the regulation of NF-κB/NOX1 signaling via interactions with IL-17RC. Notably, NOX-dependent ROS signaling is reported to accelerate the proliferation and survival of gastric epithelial cells [30]. Moreover, NF-κB activation is reportedly important for the acquisition of stemness through dedifferentiation of intestinal epithelial cells [55] and NF-κB-induced NOX1 activation promotes gastric tumorigenesis through increasing gastric stem cell proliferation [30]. In our experiments, suppression of NOX1 with a specific inhibitor reversed rhIL-17A-induced upregulation of tumor cell growth, ROS production, and stemness in AGS cells. These results are consistent with a previous study showing that pharmacological inhibition of NOX1 activity significantly suppresses gastric hyperplasia and decreases the number of SOX2-positive cells in K19-C2mE mice [30]. Although our results suggest IL-17A enhances *H. pylori*-induced gastric carcinogenesis via IL-17RC/NF-kB/NOX1 pathway, further in vivo study with inhibitors of NF-kB and/or NOX1 is needed to clarify whether this pathway is required for gastric tumorigenesis.

## Conclusion

Ablation of IL-17A clearly suppressed *H. pylori*-associated gastric carcinogenesis in our mouse model. Our findings indicate that IL-17A promotes gastric cancer growth, oxidative stress, and CSC stemness by regulating the IL-17RC/NF-κB/NOX1 pathway. The oncogenic roles of IL-17A in GC development support its potential as a therapeutic target in strategies to prevent and treat human GC.

## Supplementary Information

Below is the link to the electronic supplementary material.

Supplementary file1 (DOCX 6224 KB)
