# Supplementary material for: IL-17A promotes Helicobacter pylori-induced gastric carcinogenesis via interactions with IL-17RC
Source: Gastric Cancer. 2022 Sep 20;26(1):82–94. doi: 10.1007/s10120-022-01342-5 (PMC9813207; doi:10.1007/s10120-022-01342-5)
Supplement: Supplementary file 1 — Supplementary file1 (DOCX 6224 KB) [file 10120_2022_1342_MOESM1_ESM.docx]

**Supplemental Data to:**

**IL-17A promotes *Helicobacter pylori*-induced gastric carcinogenesis via interactions with IL-17RC**

Jee Hyun Kang^1^, Suyoung Park^1^, Jinhyung Rho^1^, Eun-Ju Hong^1^, Young-Eun Cho^2^, Young-Suk Won^3^, and Hyo-Jung Kwon^1^

*^1^Department of Veterinary Pathology, College of Veterinary Medicine, Chungnam National University, Daejeon, Korea; ^2^* *Department of Food and Nutrition, Andong National University, Andong, Korea; ^3^Laboratory Animal Resource Center, Korea Research Institute of Bioscience and Biotechnology, Chungbuk, Korea*

Jee Hyun Kang and Suyoung Park share co-first authorship.

Young-Suk Won and Hyo-Jung Kwon contribut equally and are co-corresponding authors.

**Supplementary Materials and Methods**

**Tissue microarray-based immunohistochemical staining of IL-17RC in human GC tissues**

Tissue-arrayed slides containing 140 human gastric cancer and 10 normal gastric tissues were purchased from US Biomax (MD, USA, https://www.biomax.us). All cancer tissues were categorized based on the TNM classification scheme. Immunohistochemistry was conducted using anti-IL-17RC (Atlas Antibodies, STH, Sweden) and expression of IL-17RC analyzed at 100 X original objective magnification. The study was approved by the Chungnam National University Institutional Review Board (Approval Number: 202205-BR-062-01).

**Experimental mice**

IL-17A KO mice were kindly provided by Dr. Won-Il Jeong (Korea Advanced Institute of Science and Technology, Daejeon, Korea). These mice were maintained with C57BL/6 strain and backcrossed with C57BL/6 for more than 10 generations. Animals used in this study were viral antibody-free for 14 murine viruses and negative for pathogenic bacteria (including *Helicobacter* spp.) as well as parasites. All experiments were approved by the Chungnam National University Animal Care and Use Committee (Approval Number: CNU-00598) and conducted in accordance with the Guide for Care and Use of Laboratory Animals, Institute for Laboratory Animal Research (ILAR, USA).

**Chemical treatments and bacterial infection**

*N*-methyl-*N*-nitrosourea (MNU; Sigma-Aldrich, MO, USA) solutions were freshly prepared twice a week by dissolving 150 ppm MNU in distilled water. Where indicated, solutions were administered *ad libitum* as drinking water in light-shielded bottles. Mouse-adapted *H. pylori* Sydney strains (SS1) (cagA^+^ and vacA^+^) were inoculated on Brucella agar plates (Becton Dickinson, MD, USA) containing 10% heat-inactivated fetal bovine serum (FBS) and Skirrow medium (Difco, MI, USA). *H. pylori* was maintained at 37°C under microaerobic conditions using GasPak jars (Difco) and Campy-Paks (Becton Dickinson). After 24 h of fasting, a 0.1 mL suspension of *H. pylori* (1 × 10^9^ colony-forming units (CFU)/mL) was administered to mice via intragastric intubation.

**Cell culture experiments**

The stomach cancer cell lines AGS (American Type Culture Collection, VA, USA) and SNU 601 (Korean Cell Line Bank, Seoul, Korea) were used. Cells were cultured in RPMI1640 containing 10% FBS, 100 U/ml penicillin and 100 U/ml streptomycin (Gibco, NY, USA) at 37°C and 5% CO_2_. Cells displaying arrested growth in medium supplemented with 1% fetal bovine serum (FBS) for 12 h were treated with different concentrations of recombinant human IL-17A (rhIL-17A; R&D Systems, MN, USA) over a range of time-points. According to previous studies, IL-17RC neutralizing antibody used to determine extracellular inhibition [1, 2]. Cells were plated in 6-well plates at a density of 5 X 10^5^ cells/well in RPMI1640 containing 10% FBS, pretreated with anti-IL-17RC antibody (10 µg/mL) for 1 h under serum-free conditions, and subsequently stimulated with rhIL-17A (50 ng/mL) for 2 h. Results were compared to those obtained from untreated controls and groups stimulated with rhIL-17A (50 ng/mL) alone. For inhibitor experiments, cells were pretreated with IMD-0354, a novel inhibitor of IKKβ (Tocris Bioscience, Bristol, UK) and the NOX1/4-specific inhibitor GKT136901 (Calbiochem, CA, USA) at a dose of 10 nM for 6 h.

**Histopathologic examination**

After overnight fasting, mice were euthanized. The stomachs were removed immediately and incised along the greater curvature, and the number and length (diameter) of stomach nodules recorded. Each excised stomach was cut into six strips and fixed in 10% neutral-buffered formalin (NBF). After embedding in paraffin, samples were sectioned at 4 μm thickness followed by staining with hematoxylin and eosin (H&E). Neoplastic nodule was classified using both histopathological and cytological criteria according to a previous report by our group [3] Following diagnosis, tumor incidence and multiplicity were calculated. The extent of inflammation, oxyntic gland atrophy, mucous metaplasia, foveolar hyperplasia, intestinal metaplasia and dysplasia were scored on an ascending scale from 0 to 4 based on previously outlined criteria [3].

**Immunohistochemistry and immunofluorescence**

Immunohistochemical analysis was performed using specific antibodies against IL-17A (Abcam, MA, USA) and proliferating cell nuclear antigen (PCNA; Cell Signaling, MA, USA). Vectastain ABC (Vector laboratories, CA, USA) and 3,3'-diaminobenzidine (DAB) substrate (Vector laboratories) kits were used and sections were counterstained with hematoxylin. For immunofluorescence experiments, sections were incubated with primary antibodies against phospho-nuclear factor kappa B (NF-κB; Cell Signaling, Santa Cruz biotechnology, TX, USA), NADPH oxidase 1 (NOX1; Novus Biologicals, CO, USA), CD44 (Abcam), SOX9 (Abcam), CD8 (Invitrogen, CA, USA), CD4 (Invitrogen), and Ly6G (Invitrogen), along with Alexa Fluor 488-conjugated goat anti-rabbit IgG, Alexa Fluor 555-conjugated goat anti-mouse IgG and Alexa Fluor 555-conjugated goat anti-rat IgG (Invitrogen) secondary antibodies. Ten fields (final magnification, × 400) were randomly selected for each sample and the number of positive and total cells counted using light and fluorescent microscopy, respectively (Nikon, Tokyo, Japan).

**Terminal deoxynucleotidyl transferase-mediated dUTP-biotin nick end labeling (TUNEL)**

The TUNEL assay was performed to evaluate apoptosis of gastric epithelial cells. The ApopTag Plus Peroxidase *in situ* Apoptosis Detection kit (Merck Millipore Corporation, MA, USA) was employed according to the manufacturer’s protocol. TUNEL-positive cells were counted from ten random non-overlapping fields for each section under a fluorescence microscope (Nikon) and expressed as a percentage of total cells.

**Assay of intracellular ROS**

ROS generation was assessed using 2′,7′-dichlorodihydrofluorescein (DCFH; Sigma Aldrich), which is oxidized to fluorescent dichlorofluorescein (DCF) in the presence of ROS. Cells were seeded at a density of 2.5 x 10^4^ cells/well in black 96‑well plates. After 24 h, cells were treated with rhIL-17A (50ng/ml) for 12 h. During the last hour of rhIL-17A treatment, DCFH (10µM) was added to the medium. Cells were washed three times with Dulbecco's phosphate-buffered saline (DPBS) and fluorescence intensity detected at 485 nm excitation and 535 nm emission relative to the untreated control group. To visualize DCF fluorescence, cells were cultured on 8-well chamber slides (Nalge Nunc International, IL, USA) at a density of 5 x 10^4^ cells/well. After stimulation with rhIL-17A and DCFH, cells were incubated for 1 min in 300 nM DAPI solution (Thermo Fisher Scientific, MA, USA) and washed with DPBS. Finally, the 8-chamber upper structure was removed, mounting solution added and a coverslip applied. To examine ROS levels in the stomach, tissue sections were incubated with DCFH in a humidified chamber with light avoidance for 30min at 37℃. DCF-positive cells were detected under a fluorescence microscope (Nikon). Five random epithelial fields for each section were selected for quantification and DCF-positive areas measured using Image J software.

**Formation of tumor sphere**

Cells were seeded in ultra-low attachment dishes and cultured in conditioned DMEM/F12 stem cell medium (Gibco) supplemented with 20 ng/mL epidermal growth factor (EGF; Invitrogen), 20 ng/mL basic fibroblast growth factor (bFGF; Invitrogen) and B27 supplement (Invitrogen). The culture medium was replaced or supplemented with additional growth factors every 4 days. For the sphere formation assay, cells were treated with rhIL-17A and NOX1/4-specific inhibitor, GKT13690, and the total number of tumor spheres counted following 4 weeks of culture.

**Flow cytometry**

Flow cytometry was performed on BD FACSCanto™ System (BD Bioscience, CA, USA). For analysis of cell cycle distribution, cells were fixed in 70% ethanol and stained with FxCycle^TM^ PI/RNase Staining Solution (Thermo Fisher Scientific) following the manufacturer’s protocol. The percentage (%) of cells with DNA contents representing the G0/G1, S and G2/M phases was analyzed using BD FACSDiva™ software. For detection of apoptosis, cells were stained using FITC Annexin V Apoptosis Detection Kit I (BD Bioscience) according to the manufacturer’s instructions and the percentages (%) of apoptotic cells calculated.

**Western blot analysis**

Cells or stomach (n = 3-14 mice per group) were lysed in RIPA lysis buffer (Cell Signaling) supplemented with a protease inhibitor cocktail (Roche Diagnostics, Basel, Switzerland). Equal amounts of proteins were mixed with the same volume of 2x sample buffer (Bio-Rad Laboratories, CA, USA) and boiled for 10 minutes. All samples were subjected to SDS-PAGE with 8 % gels. Separated proteins were transferred onto polyvinylidene fluoride (PVDF) membrane (Merck Millipore), followed by blocking in 5% nonfat dry milk and blotting with primary antibodies against IL-17A, IL-17RA, IL-17RC, cyclin D1, phospho-NF-κB, total-NF-κB, and NOX1. After washing, the membrane was incubated with HRP-conjugated secondary antibodies according to the manufacturer’s instructions. The SuperSignal West Femto Maximum Sensitivity Substrate (Thermo Fisher Scientific) was used for visualization. Expression of different proteins was detected using the EzWestLumi plus system (ATTO Corporation, Tokyo, Japan), and quantified using CSAnalyzer 4 relative to β‐actin.

**RNA extraction and real-time PCR**

Total RNA was isolated from cells using a RNeasy Micro Kit (Qiagen, Hilden, Germany) according to the manufacturer’s instructions. An aliquot of total RNA (1 µg) obtained from each sample was reverse-transcribed into cDNA with oligo (dT) primers using a ReverTra Ace™ qPCR RT Kit (Toyobo, Tokyo, Japan). Reaction mixtures were amplified with SYBR Green PCR Master Mix (Applied Biosystems, CA, USA) using the primers listed in Supplementary Table 1. Fluorescence intensity was measured in real-time during the extension step using the Applied Biosystems 7500 Real-Time PCR System (Life Technologies, CA, USA). Relative expression levels of the target gene were normalized using GAPDH as an endogenous reference based on the comparative CT (threshold cycle) method.

**Analysis of IL-17RC immunohistochemical staining in human GC tissues**

Staining scores for each protein were obtained by multiplying the percentage of positively stained cells/areas by intensity of staining. Positive immunoreactivity scores were categorized as follows: 0: <5% stained cells/area, 1: 5-25% stained cells/area, 2: 26-50% stained cells/area, 3: 51-80% stained cell/area, and 4: ≥ 81% stained cells/area. Staining intensity was evaluated using the following criteria: 0 (negative), 1 (week), 2 (moderate) and 3 (strong). Staining grades were scored 0–3 obtained by multiplying the area by intensity (negative: 0, 1, or 2; weak: 3, 4, or 5; moderate: 6, 7, or 8; strong: 9, 10, 11, or 12).

**Supplementary Fig. 1**


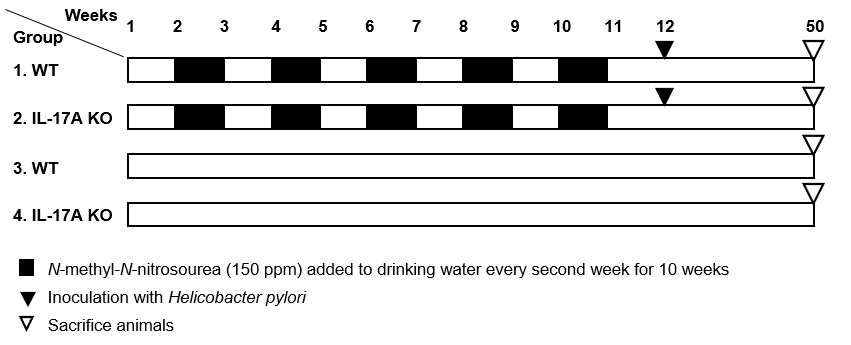


**Supplementary Fig. 1 Study design.** Mice were randomized into four groups according to treatments, specifically, Groups 1 and 2 (5 weeks of MNU administration followed by *H. pylori* infection) and Groups 3 and 4 (control). Animals were sacrificed at 38 weeks after *H. pylori* infection.

**Supplementary Fig. 2**

**
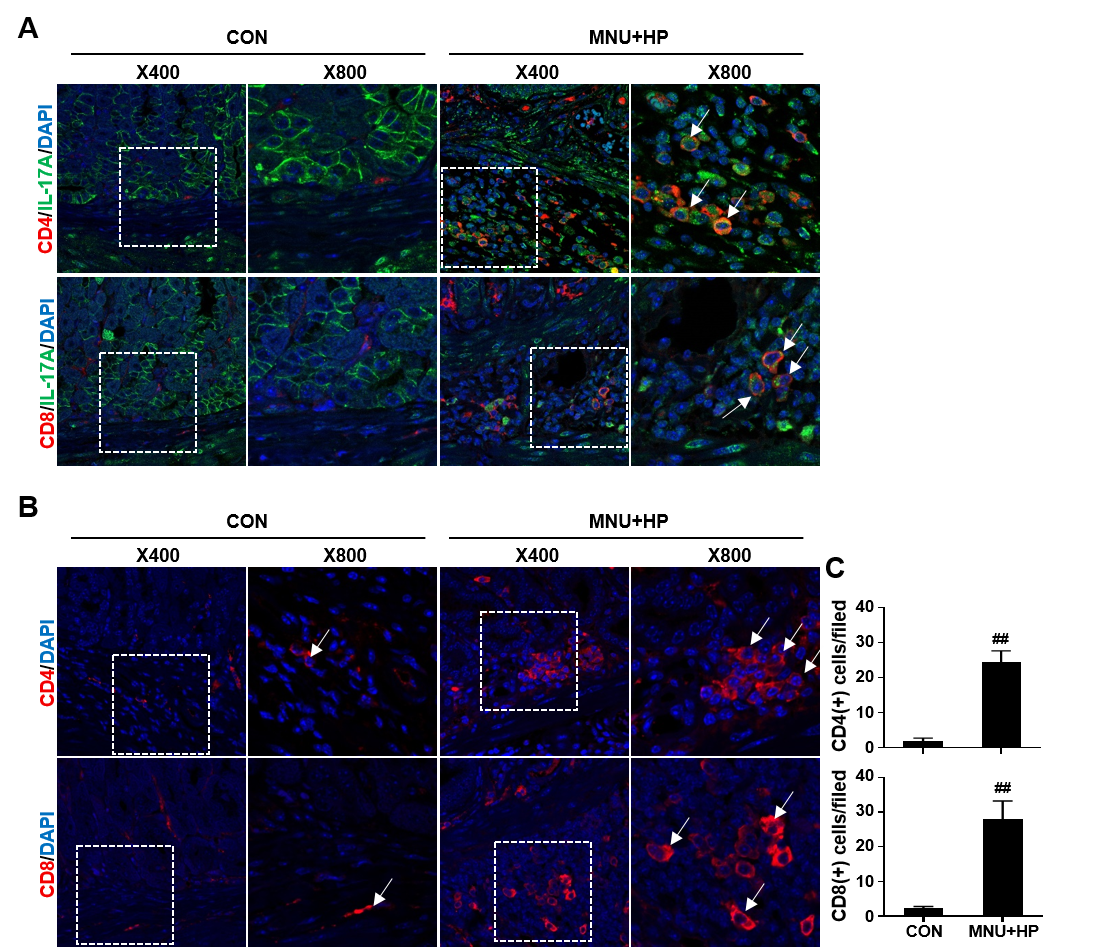
**

**Supplementary Fig. 2 Immune-cell staining in stomach tissues of WT mice.** (A) Representative images of IL-17A (green), CD4 (red), and CD8 (red) in submucosa from mice treated with and without MNU and *H. pylori*. Note the co-staining of IL-17A and CD4 or CD8 (white arrows). (B) Immunofluorescence staining of CD4 and CD8 in control and MNU and *H. pylori*-treated mice. Boxed regions of left panels (original magnification = X400) are shown at higher magnification in the right panels (original magnification = X800). (C) The number of CD4 or CD8 positive cells. Data are expressed as mean ± SEM. **P* < 0.05, ***P* < 0.01 versus control.

**Supplementary Fig. 3**


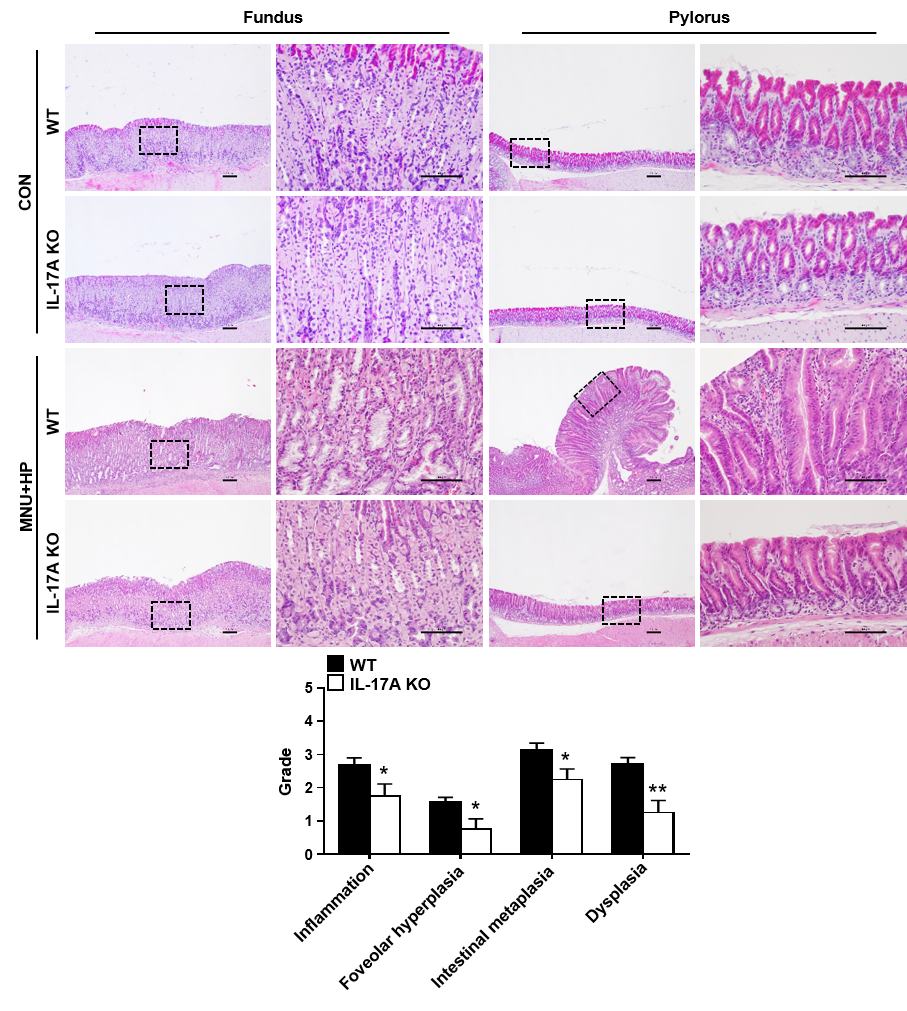


**Supplementary Fig. 3 Gastric histopathology of WT and IL-17A KO mice.** Histological sections of fundus and antrum from control WT and IL-17A KO mice stained with H&E. Oxyntic atrophy and functional loss of parietal cell mass were observed due to mucous metaplasia characterized by foamy changes of the cytoplasm of parietal cells in fundus of MNU and *H. pylori-*treated WT mice. Inflammatory cell infiltration was evident in the lamina propria and submucosa, with mucosal hyperplasia and intestinal metaplasia characterized by epithelial cell columnar elongation and rare goblet cells in antrum of MNU and *H. pylori-*treated WT mice. Boxed regions of left panels (original magnification = X40) are shown at higher magnification in the right panels (original magnification = X200). **P* < 0.05, ***P* < 0.01 versus WT.

**Supplementary Fig. 4**

**
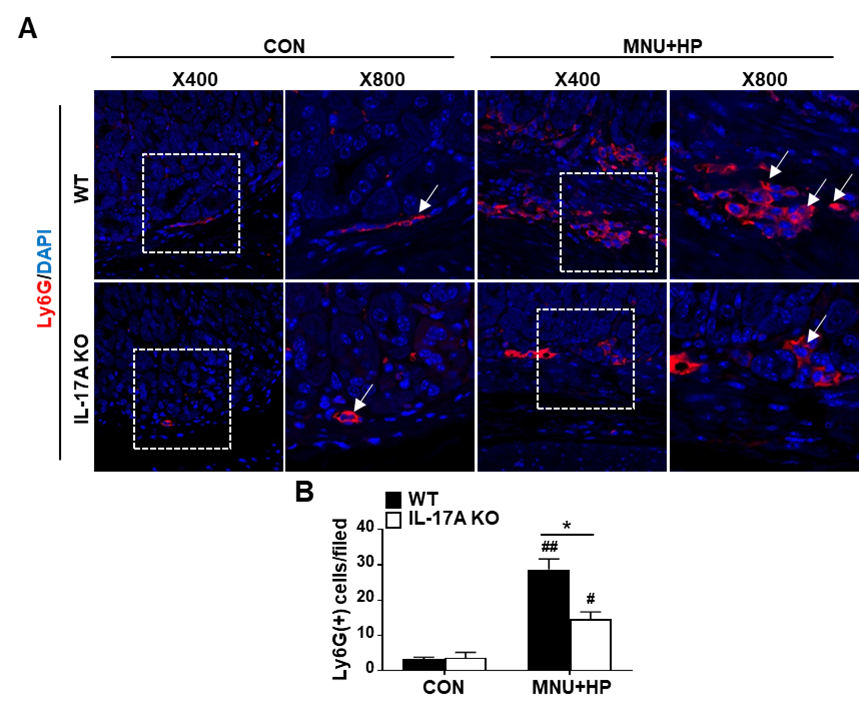
**

**Supplementary Fig. 4 Immunofluorescence analysis of Ly-6G.** (A) Immunofluorescence staining of Ly6G in the stomach of WT and IL-17A KO mice. Boxed regions of left panels (original magnification = X400) are shown at higher magnification in the right panels (original magnification = X800). (B) The number of Ly6G positive cells. Data are expressed as mean ± SEM. ^*^*P* < 0.05 versus WT; ^#^*P* < 0.05, ^##^*P* < 0.01 versus same genotype control.

**Supplementary Fig. 5**

**
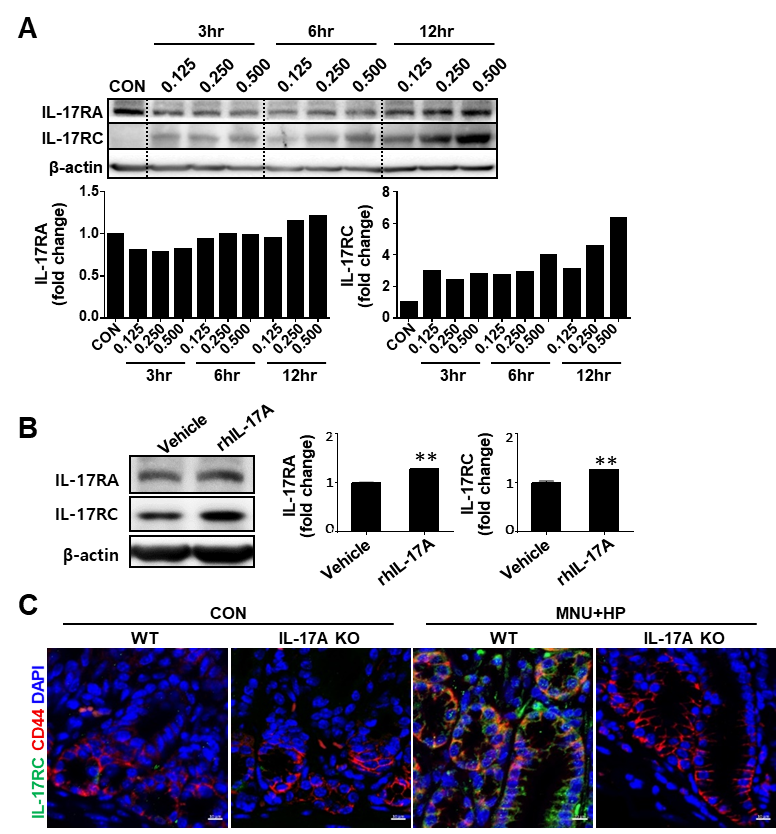
**

**Supplementary Fig. 5 IL-17A enhances *H. pylori*-induced gastric carcinogenesis via interaction with IL-17RC.** (A) Western blot analysis of IL-17RA and IL-17RC in *H. pylori*-treated AGS cells. (B) Immunoblot analysis of IL-17RA and IL-17RC in rhIL-17A-treated SNU601 cells. (C) Double immunofluorescence of IL-17RC (green) and CD44 (red) in the stomach of WT and IL-17A KO mice. Original magnification = X400. Data are expressed as mean ± SEM. ^*^*P* < 0.05, ^**^*P* < 0.01 versus vehicle.

**Supplementary Fig. 6**

**
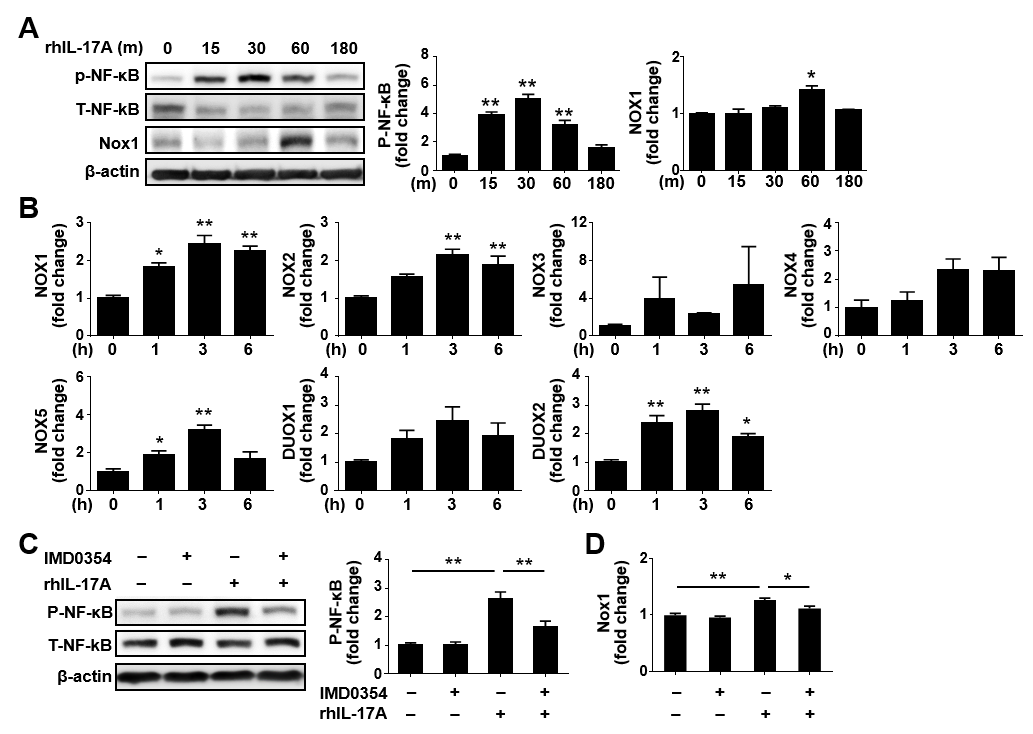
**

**Supplementary Fig. 6 IL-17A activates NF-kB/NOX1 pathway. (**A) Western blot analysis of phospho-NF-κB and NOX1 in rhIL-17A-treated SNU 601 cells. (B) Real-time PCR of NOX transcripts in AGS cells treated with rhIL-17A. (C) Western blot analysis of phospho-NF-κB in AGS cells treated with rhIL-17A and/or IMD0354. (D) Real-time PCR analysis of NOX1 expression in AGS cells treated with rhIL-17A and/or IMD0354. Data are presented as mean ± SEM. ^*^*P* < 0.05, ^**^*P* < 0.01 versus 0 h or vehicle.

**Supplementary Fig. 7**

**
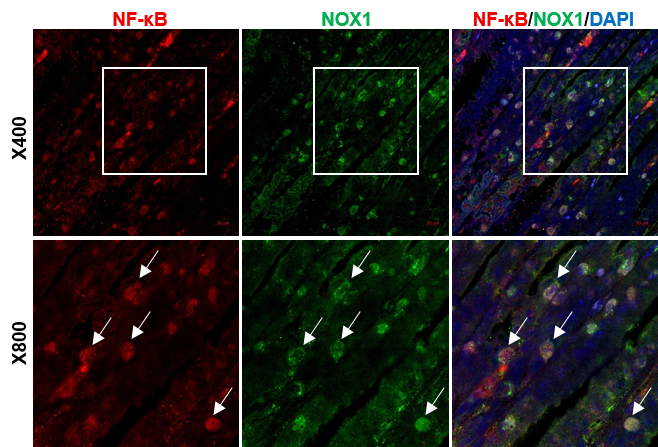
**

**Supplementary Fig. 7 Immunofluorescence staining of phospho-NF-κB and NOX1.** Double immunofluorescence of phospho-NF-κB (red) and NOX1 (green) in the gastric mucosa of MNU and *H. pylori* treated mice. Note the co-localization of phospho-NF-κB and NOX1 (arrows). Original magnification = X400 (upper) and X800 (lower).

**Supplementary Table 1.** Primers used to amplify target genes

| **Gene** | **Primer** |
| --- | --- |
| *DUOX1* | Forward: 5'-CTTGAACAATTTGTGCGGCT-3'  Reverse: 5'-GCAGGGTGGTATTTCGGATT-3' |
| *DUOX2* | Forward: 5'-GGGTTTTCAAAGCTCCCAAG-3'  Reverse: 5'- TGT TGT CCT CCA ACT CCG AA-3' |
| *E-cadherin* | Forward: 5'-GCCTCCTGAAAAGAGAGTGGAAG-3'  Reverse: 5'- TGGCAGTGTCTCTCCAAATCCG-3' |
| *Fibronectin* | Forward: 5'-ACAACACCGAGGTGACTGAGAC-3'  Reverse: 5'-GGACACAACGATGCTTCCTGAG-3' |
| *Lgr5* | Forward: 5'-TTGCTTCAGCTCCACAGAGA-3'  Reverse: 5'-TGGTTGTAGAGGGCAAGGAC-3' |
| *NOX1* | Forward: 5'-AATGCTGTCACCGATATTCCA-3'  Reverse: 5'-AGATTACCGTCCTTATTCCTATAACTC-3' |
| *NOX2* | Forward: 5'-TGTTAGTGGGAGCAGGGATTG-3'  Reverse: 5'-TCAGATTGGTGGCGTTATTGC-3' |
| *NOX3* | Forward: 5'-CACTGAAGCTGAGCAAGGTGTA-3'  Reverse: 5'-CGTGTTTCCAGGGAGAGTAAGAGA-3' |
| *NOX4* | Forward: 5'-GCATGTGGCTGCCCATCT-3'  Reverse: 5'-GCCAGGAACAGTTGTGAAGAGA-3' |
| *NOX5* | Forward: 5'-TGGATCGAAGGTGTCCAAGA-3'  Reverse: 5'-CAGCAGGCTCACAAACCACT-3' |
| *Vimentin* | Forward: 5'-AGGCAAAGCAGGAGTCCACTGA-3'  Reverse: 5'-ATCTGGCGTTCCAGGGACTCAT-3' |

**Supplementary Table 2.** Incidence and multiplicity of MNU and *Helicobacter* pylori-associated gastric tumor

| Group | IL-17A genotype | MNU+HP | Total No  of mice | No of tumor bearing mice  (% incidence) | Gastric adenoma (%) | Gastric adenocarcinoma (%) | Tumor multiplicity |
| --- | --- | --- | --- | --- | --- | --- | --- |
| 1 | WT | Yes | 14 | 12 (85.71) | 4 (28.57) | 8 (57.14) | 0.86±0.10 |
| 2 | KO | Yes | 9 | 2 (22.22)** | 01 (11.11) | 1 (11.11)* | 0.22±0.13* |
| 3 | WT | No | 9 | 0 | 0 | 0 | 0 |
| 4 | KO | No | 8 | 0 | 0 | 0 | 0 |

Data are presented as means ± S.E.M. **P* < 0.05 *vs* respective WT mice.

**Supplementary references**

1. Zrioual S, Toh M-L, Tournadre A, Zhou Y, Cazalis M-A, Pachot A, et al. IL-17RA and IL-17RC receptors are essential for IL-17A-induced ELR+ CXC chemokine expression in synoviocytes and are overexpressed in rheumatoid blood. The Journal of Immunology. 2008;180(1):655-63.
2. Chang Y, Al-Alwan L, Risse P-A, Roussel L, Rousseau S, Halayko AJ, et al. TH17 cytokines induce human airway smooth muscle cell migration. J Allergy Clin Immunol. 2011;127(4):1046-53. e2.
3. Kwon H-J, Won Y-S, Nam K-T, Yoon Y-D, Jee H, Yoon W-K, et al. Vitamin D3 upregulated protein 1 deficiency promotes N-methyl-N-nitrosourea and Helicobacter pylori-induced gastric carcinogenesis in mice. Gut. 2012; 61: 53-63.
